# Simultaneous BOLD-fMRI and constant infusion FDG-PET data of the resting human brain

**DOI:** 10.1038/s41597-020-00699-5

**Published:** 2020-10-21

**Authors:** Sharna D. Jamadar, Phillip G. D. Ward, Thomas G. Close, Alex Fornito, Malin Premaratne, Kieran O’Brien, Daniel Stäb, Zhaolin Chen, N. Jon Shah, Gary F. Egan

**Affiliations:** 1 Monash Biomedical Imaging, Monash University, Melbourne, VIC Australia; 2 Australian Research Council Centre of Excellence for Integrative Brain Function, Melbourne, Australia; 3 Turner Institute for Brain and Mental Health, Monash University, Melbourne, VIC Australia; 4 Australian National Imaging Facility, Brisbane, QLD Australia; 5 Department of Electrical and Computer Systems Engineering, Monash University, Melbourne, VIC Australia; 6 Siemens Healthineers, Siemens Healthcare Pty Ltd, Bayswater, VIC 3153 Australia; 7 Institute of Neuroscience and Medicine, Forschungszentrum Jülich, 52425 Jülich, Germany

**Keywords:** Neural circuits, Network models

## Abstract

Simultaneous [18 F]-fluorodeoxyglucose positron emission tomography and functional magnetic resonance imaging (FDG-PET/fMRI) provides the capability to image two sources of energetic dynamics in the brain – cerebral glucose uptake and the cerebrovascular haemodynamic response. Resting-state fMRI connectivity has been enormously useful for characterising interactions between distributed brain regions in humans. Metabolic connectivity has recently emerged as a complementary measure to investigate brain network dynamics. Functional PET (fPET) is a new approach for measuring FDG uptake with high temporal resolution and has recently shown promise for assessing the dynamics of neural metabolism. Simultaneous fMRI/fPET is a relatively new hybrid imaging modality, with only a few biomedical imaging research facilities able to acquire FDG PET and BOLD fMRI data simultaneously. We present data for n = 27 healthy young adults (18–20 yrs) who underwent a 95-min simultaneous fMRI/fPET scan while resting with their eyes open. This dataset provides significant re-use value to understand the neural dynamics of glucose metabolism and the haemodynamic response, the synchrony, and interaction between these measures, and the development of new single- and multi-modality image preparation and analysis procedures.

## Abstract (summary)

**Table:** 

Measurement(s)	glucose import • haemodynamic response • functional brain measurement
Technology Type(s)	FDG-Positron Emission Tomography • Blood Oxygen Level-Dependent Functional MRI
Factor Type(s)	resting human brain
Sample Characteristic - Organism	Homo sapiens

Machine-accessible metadata file describing the reported data: 10.6084/m9.figshare.12994508

## Background & Summary

Simultaneous magnetic resonance imaging - positron emission tomography (MRI-PET) represents a significant development in human imaging neuroscience. Simultaneous BOLD-fMRI/FDG-PET (blood oxygen level-dependent functional magnetic resonance imaging/[18 F]-fluorodeoxyglucose positron emission tomography) enables the simultaneous measurement of the two of the most widely used *in vivo* markers of neuronal activity. The simultaneous nature of the acquisition is of particular importance, as it enables glucose uptake and haemodynamic responses to the same neuronal activity to be captured, without the confounds of intra-individual differences in attention, fatigue, motivation, nutrient intake and blood chemistry that occur in non-contemporaneous acquisitions^1^.

BOLD-fMRI provides a haemodynamic-based surrogate of neuronal activity with a temporal resolution of the order of seconds and spatial resolution of around a millimetre. The BOLD signal is comprised of both neuronal and non-neuronal components. The neuronal component of the BOLD signal is believed to arise from local field potentials of peri-synaptic activity^2,3^, whereas the non-neuronal component appears from cerebrovascular sources, including cerebral blood flow, volume, and the metabolic rate of oxygen. The BOLD signal, therefore, is confounded by intra- and inter-individual differences in heart rate variability, respiration, haemoglobin concentration and the oxygen-carrying capacity of the blood^4–8^.

BOLD-fMRI is a semi-quantitative index of neuronal function that cannot be compared across brain regions, subjects, or imaging sites^3^. By comparison, FDG-PET is a fully quantitative index of neuronal activity that captures cerebral glucose uptake that is primarily localised to the synapses^9–11^. FDG-PET has a spatial resolution of around 4 mm^12^ and, until recently, a temporal resolution that was effectively equal to the scan duration – around 10–40 minutes. However, recent developments in radiotracer delivery^13,14^ have resulted in substantial improvements in FDG-PET temporal resolution, reducing it down to 60 sec^14–18^ or less [12 sec^19^; 16 sec^13,20^; 30 sec^21^]. This method, known as ‘functional’ PET (fPET) administers the radiotracer throughout the scan, either as a constant infusion^14^ or hybrid bolus/infusion^19^. Detailed protocols for continuous infusion and hybrid bolus/infusion radiotracer administration are provided in^13^.

Functional connectivity measures the temporal coherence of neural signals across distributed regions of the brain. BOLD-fMRI has been incredibly useful for characterising the integrative activity of brain networks distributed across the brain^22,23^. Canonical resting-state networks include the default mode, dorsal attention, salience networks, and others^23^. This method is commonly labelled resting-state *functional* connectivity, but here we use the term *haemodynamic* connectivity, as functional connectivity can be measured by any functional neuroimaging method, including EEG^24^, PET^25^, fNIRS^26^, etc. Resting-state connectivity measured using static FDG-PET predates resting-state fMRI by at least a decade^25^. However, due to the limited temporal resolution of traditional FDG-PET, resting-state ‘connectivity’ was estimated as the covariance of FDG-PET signals across-subjects, rather than the temporal coherence of brain signals within a subject, as is the case in haemodynamic connectivity. Therefore, we use the term ‘metabolic covariance’ to refer to region-to-region, static, across-subject FDG-PET correlation from now on. With the development of fPET methodology, it is now possible to estimate the intra-subject dynamics of glucose uptake during the resting-state – that is, ‘metabolic connectivity’. Using this data, we^20^ have shown that resting-state fPET metabolic connectivity is similar to BOLD-fMRI haemodynamic connectivity in the frontoparietal cortex and dissimilar in other regions of the brain (subcortical, temporo-occipito regions). Furthermore, fPET metabolic connectivity was dissimilar to static FDG-PET metabolic covariance across the brain. This work forms the basis of a new field for human imaging neuroscience – the characterisation and exploration of anatomically distributed networks of coupled metabolic dynamics.

**Fig. 1:**
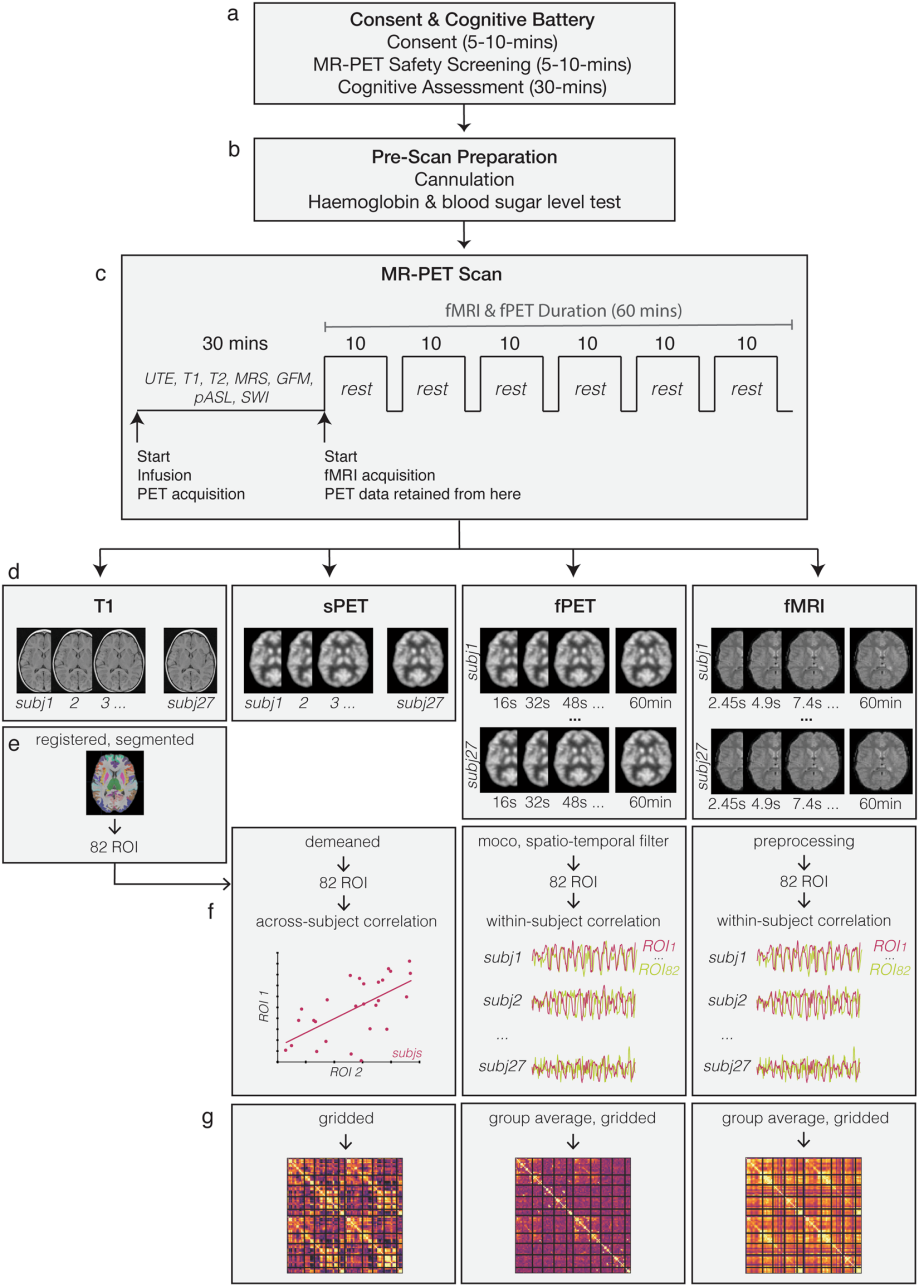
Paradigm & workflow. Panels a–d indicate the workflow for the data available in the Monash rsPET-MR dataset^27^ and panels e-g indicate the workflow for the results presented under Technical Validation, and in Jamadar *et al*.^20^. (**a**) Participants completed a demographics, safety screening and cognitive assessment an hour prior to MR-PET scanning. (**b**) Next, participants were prepared for scanning; a cannula was placed in the forearm vein of each arm, and then haemoglobin and blood sugar level was taken. (**c**) Participants then underwent a 95-minute MR-PET scan using the paradigm shown here. (**d**) Illustration of the data obtained for each method (left to right): Structural T1 MRI anatomical images for each subject (subj); static PET (sPET) acquired a single image per subject; functional PET (fPET) was binned into 16 sec images, resulting in a timeseries of images for each subject; fMR images were obtained with TR 2.45 sec, resulting in a timeseries for each subject. (**e**) Structural T1 MRI was registered to MNI space and then segmented into 82 regions of interest (ROI). This parcellation was applied to the sPET, fPET and fMRI images. (**f**) Illustration of example processing steps for each modality, as used in Jamadar *et al*. (2020). sPET was demeaned, parcellated into 82 regions (ROIs) and then correlated across subjects. Scatterplot shows an example correlation between two regions across subjects, out of a total of 82 × 82 region-wise correlations. fPET was motion corrected (moco), filtered, parcellated, and then correlated across time-series for each subject. Illustration shows 2 example timeseries of the total 82 × 82 region-by-region correlations conducted. fMRI was preprocessed, parcellated, correlated across time for each subject, then group-averaged. (**g**) Subject-level matrices for sPET, fPET and fMRI were then group averaged. Matrices are indicative of potential connectivity matrices, and are those that are reported in Jamadar *et al*.^20^.

Here, we describe the **Monash rsPET-MR** dataset^27^: a simultaneous fMRI-fPET dataset acquired from young, healthy individuals at rest (Fig. 1). We make this data publicly available as few biomedical imaging facilities currently have the requisite technology to acquire similar data. The Monash rsPET-MR dataset has re-use value to explore the dynamics of glucose metabolic and haemodynamic signals across distributed regions of the brain. The dataset also has re-use value to support the development of new imaging processing and analysis strategies for simultaneous fMRI/fPET. Simultaneous MRI-PET, fMRI-fPET, and the fPET methodology are nascent technologies with immature processing pipelines. As new methods, these fields do not benefit from many years of work validating data preparation and signal detection optimisation, particularly in comparison to the fMRI field. Examples of important validation work yet to be performed includes test-retest reliability (which can be difficult in humans for ethical reasons due to biosafety constraints related to radioactivity exposure), and whether the FDG in a continuous infusion protocol acts as a radiotracer or is more analogous to a radioligand^28^. Examples of re-use of the Monash rsfPET-MR dataset may include development of data analysis techniques^18^, synergistic data fusion techniques^29^, discoveries about the relationship between glucose uptake and the haemodynamic response^17^, as well as revelations about the fundamental basis of energy use in the human brain^21,30–32^.

## Methods

All methods were reviewed by the Monash University Human Research Ethics Committee, following the Australian National Statement of Ethical Conduct in Human Research (2007). Subjects provided informed consent to participate in the study. Administration of ionising radiation was approved by the Monash Health Principal Medical Physicist, following the Australian Radiation Protection and Nuclear Safety Agency Code of Practice (2005). For participants aged over 18-yrs, the annual radiation exposure limit of 5 mSv applies, and the effective dose in this study was 4.8 mSv.

A video methods article describing the constant infusion acquisition procedure is reported in^13^. A comparison of static PET, fPET, and fMRI connectivity using this data is reported in^20^. The dataset reported here includes demographic information, anthropometry, and minimally-processed simultaneously acquired MRI and PET images acquired during the course of a more extensive study.

### Participants

Participants (n = 27) were aged 18–23 years (mean 19 years), 21 female, all right-handed. Participants had between 13–18 years of education (mean 14 years), normal or corrected-to-normal vision, and no personal history of diagnosed Axis-1 mental illness, diabetes, or cardiovascular illness. Participants were screened for claustrophobia, non-MRI compatible implants, clinical, or research PET scan in the previous 12 months. Women were screened for current or suspected pregnancy.

### Procedure

Figure 1 presents the acquisition and analysis workflow for the study.

Before the scan, participants were directed to consume a high protein/low sugar diet for 24hrs, fast for 6hrs, and drink 2–6 glasses of water. Participants completed a demographic assessment (10-min) and a brief cognitive battery (30-mins, data not reported here).

Participants were cannulated in a vein in each forearm with a minimum size 22-gauge cannula, and a 10 mL baseline blood sample was taken at the time of cannulation. For all participants, the left cannula was used for FDG infusion, and the right cannula was used for blood sampling. Primed extension tubing was connected to the right cannula (for blood sampling) via a three-way tap.

Participants underwent a 95-minute simultaneous MR-PET scan in a Siemens (Erlangen) 3 Tesla Biograph molecular MR (mMR) scanner (Syngo VB20 P). Quality control is performed daily on the scanner using a 68-Ge (germanium) phantom. Cross-calibration is also conducted between the scanner, dose calibrator and well counter on delivery of the radiotracer dose using an 18-F and water phantom.

Participants were positioned supine in the scanner bore with their head in a 16-channel radiofrequency (RF) head coil, and were instructed to lie as still as possible with eyes open, and think of nothing in particular. [18-F] fluorodeoxyglucose (FDG; average dose 233MBq) was infused over the course of the scan at a rate of 36 mL/hr using a BodyGuard 323 MR-compatible infusion pump (Caesarea Medical Electronics, Caesarea, Israel). One participant received a lower dose (167MBq) due to an infusion pump error. Infusion onset was locked to the start of the PET scan.

Plasma radioactivity levels were measured throughout the duration of the scan. At 10-mins post-infusion onset, a 10 mL blood sample was taken from the right forearm using a vacutainer; the time of the 5 mL mark was noted for subsequent decay correction. Subsequent blood samples were taken at 10-min intervals for a total of 10 samples for the duration of the scan. The cannula line was flushed with 10 mL of saline after every sample to minimise line clotting. Immediately following blood sampling, the sample was placed in a Heraeus Megafuge 16 centrifuge (ThermoFisher Scientific, Osterode, Germany) and spun at 2000rpm for 5 mins; 1000 μL plasma was pipetted, transferred to a counting tube and placed in a well counter for 4 mins. The count start time, the total number of counts, and the counts per minute were recorded for each sample.

### Scanning protocol

PET data were acquired in list mode. Infusion of the FDG radiotracer and PET data acquisition started with the Ultrashort TE (UTE) MRI for PET attenuation correction. While the PET signal rose to detectable levels over the first 30-mins following infusion onset, non-functional MRI scans were acquired. These scans included^27^: T1 3D MPRAGE (TA = 7.01 mins, TR = 1640ms, TE = 2.34 ms, flip angle = 8°, FOV = 256 × 256mm^2^, voxel size = 1 × 1 × 1mm^3^, 176 slices; sagittal acquisition), gradient field map (TA = 1.02 min); and several scans not reported here: UTE (TA = 1.40 mins), T2 SPACE (TA = 5.52 min), magnetic resonance spectroscopy (MRS, TA = 2.48 min), pulsed arterial spin labelling (TA = 4.21), T2 susceptibility-weighted image (TA = 6.50 min), and left-right phase correction (TA = 0.21 min). For the remainder of the scan, six consecutive 10 min blocks of T2*-weighted echo-planar images (EPIs) were acquired (TR = 2450 ms, TE = 30 ms, FOV = 190 mm, 3 × 3 × 3mm 3 voxels, 44 slices, ascending axial acquisition).

## Data Records

Table 1 defines the demographic and imaging data available for each subject on *OpenNeuro*. The dataset containing the demographic, fMRI, PET, T1 structural and gradient field maps is freely available in BIDS format^33^ from the *OpenNeuro* repository (http://openneuro.org) with the accession number ds002898^27^.

**Table 1:** Data fields available for the Monash rsPET-MRI dataset^27^.

Field	Description	Values	Notes
participants.tsv			Demographics and anthropometry for participants
age	age of the participant	years	
sex	sex of the participant as reported by the participant	“M”: “male”, “F”: “female”	
handedness	handedness of the participant as reported by the participant	“L”: “left”, “R”: “right”	Self-report
education_years	years of education calculated as formal education of 6 months or more. starts from the first year of primary/elementary school onwards.	years	Note that Australian education system includes 13 years of school: 7 years primary/elementary 6 years high school.
education_specify	the highest level of formal education completed (i.e., does not include uncompleted education or education currently undertaken).	string	In Australia, ‘technical school’ is education undertaken in the TAFE/vocational education sector
efl	english as first language	“yes”: “Yes”, “no”: “No”	
first_language	first language spoken	string	Specified only for those responding ‘no’ to efl
visual_impairment	any kind of vision impairment including wearing reading glasses	“yes”: “Yes”, “no”: “No”	Self-report
visual_impairment_specify	description of visual impairment	string	Specified only for those responding ‘yes; to visual_impairment
mental_illness_history	self-report of whether the person has had a current or past Axis I psychiatric condition	“yes”: “Yes”, “no”: “No”	
mental_illness_specify	diagnosis or treatment of whether the person has received a diagnosis for any Axis I psychiatric condition	string	
cardiovascular_disease	history of cadiovascular disease	“yes”: “Yes”, “no”: “No”	
diabetes	history of diabetes	“yes”: “Yes”, “no”: “No”	
regular_medication	whether the person is currently taking regular medication	“yes”: “Yes”, “no”: “No”	
regular_medication_specify	description of regular medication	string	
current_smoker	currently smoking	“yes”: “Yes”, “no”: “No”	
current_smoker_specify	description of current smoking	string	
previous_smoker	previous smoking	“yes”: “Yes”, “no”: “No”	
previous_smoker_specify	description of previous smoking	string	
alcohol_consumption	self-report of alcohol consumption	“yes”: “Yes”, “no”: “No”	
alcohol_drinking_days	number of days per week/month the person drinks	numeric	
alcohol_number_drinks	number average number of standard drinks per drinking session	numeric	
alcohol_notes	description of alcohol consumption	string	
recreational_drugs	used recreational drugs in last 6 months	“yes”: “Yes”, “no”: “No”	
recreational_drugs_specify	description of recreational drugs used in last 6 months	string	
edinburgh_hand_r	Edinburgh Handedness Inventory: right-hand	numeric	Total score for right handed items
edinbugh_hand_l	Edinburgh Handedness Inventory: left-hand	numeric	Total score for left handed items
cesd_r	Centre for Epidemiological Studies Depression Inventory - Revised total score	numeric	
sub-*			Folder for each subject separately. Labelled ‘sub-01’, ‘sub-02’, etc.
anat	T1w.json Defacemask.nii.gz T1w.nii.gz	T1_mprage_sag_1_iso	T1 MPRAGE
fmap	magnitude1.json magnitude2.json phasediff.json magnitude1.nii.gz magnitude2.nii.gz phasediff.nii.gz	short TE long TE	Gradient field maps
func	task-rest_run-1_bold.json task-rest_run-2_bold.json task-rest_run-3_bold.json task-rest_run-4_bold.json task-rest_run-5_bold.json task-rest_run-6_bold.json task-rest_run-1_bold.nii.gz task-rest_run-2_bold.nii.gz task-rest_run-3_bold.nii.gz task-rest_run-4_bold.nii.gz task-rest_run-5_bold.nii.gz task-rest_run-6_bold.nii.gz	T2* EPI	T2* BOLD-fMRI EPIs for 6 blocks
pet	task-rest_pet.json defacemask.nii.gz task-rest_pet.nii.gz	16 sec recon FDG-PET data	16 sec reconstructed FDG-fPET data, attenuation corrected
derivatives			
mcflirt/sub-*/pet/sub- *_task_rest_pet_moco.par	Space-delimited realignment parameters (pitch,roll,yaw,x,y,z) for each pet frame	numeric	

Participants.tsv is a text file reporting the demographic data for each subject ordered by subject ID.

T1 structural and fMRI data along with supporting gradient field maps and face mask were organised in sub-directories according to the Brain Imaging Data Structure (BIDS)^33^. Following the BIDS structure, the sub-*/anat directory contains the T1 MPRAGE data. T1 structural data were converted from DICOM to NIfTI format with JSON sidecars using the Dcm2niix converter^34^. Facial features were removed from the T1 structural images by applying a co-registered binary face mask^35,36^. The sub-*/fmap directory contains the gradient field map data: short-TE, long-TE, and phase difference images. The sub-*/func directory contains the T2* BOLD-fMRI data. fMRI data were converted from DICOM to NIfTI format with JSON sidecars using the Dcm2niix converter^34^.

The sub-*/pet directory contains the reconstructed PET data. The 5700-second list-mode PET data for each subject were binned into 356 3D sinogram frames each at 16-second intervals. PET data were converted from DICOM to NIfTI format with JSON sidecars using the Dcm2niix converter^34^. To remove facial features from the PET data, a binary face mask was co-registered to static PET images created by summing the PET activity over the acquisition and applying to the dynamic PET data^35,36^. JSON sidecars were augmented with metadata in accordance with guidelines for the content and format of PET brain data^37^ (Knudsen *et al*.^37^) and the current working copy of the proposed BIDS Extension for PET v0.0.1 (BEP009).

PET motion-correction parameters estimated for each subject are included in accordance with the recommendations for derivatives in BIDS datasets. Space-delimited realignment parameters [6 (pitch, roll, yaw, x, y, z)] for each frame (225) are stored on separate rows of a text file at derivatives/mcflirt/sub-*/pet/sub-*_task_rest_pet.moco.par.

## Technical Validation

The Monash rsPET-MR dataset is comprised of simultaneously acquired BOLD-fMRI and FDG-fPET data acquired during the resting-state^27^. Since fPET is the primary novel outcome from this dataset, this section focuses on the technical validation of the fPET data.

To validate the data, the following processing pipeline was used (Fig. 1d–f). For the T1 structural image, the brain was extracted, then registered to MNI152 space using Advanced Normalization Tools (ANTs)^38^. The grey matter, white matter, and brain cortex labels of T1 images were segmented using Freesurfer with the Desikan-Killiany Atlas (Diedrichsen *et al*. 2009).

For the fMRI data, the six blocks of EPI scans underwent brain extraction (FSL BET, Smith, 2002), N4 bias field correction (ANTs, Tustison *et al*., 2010), motion correction (FSL MCFLIRT, Jenkinson *et al*.^36^), slice time correction (AFNI, Cox, 1996), and high pass filtering (>0.01 Hz) to remove low frequency noise (FSL, Jenkinson *et al*.). Subject motion was assessed using realignment parameters from MCFLIRT.

For the PET data, the pseudoCT method^39^ was used to correct the attenuation for all acquired data. The Ordinary Poisson-Ordered Subset Expectation Maximization (OP-OSEM) algorithm (3 iterations, 21 subsets) with point spread function correction was used to reconstruct 3D volumes from the sinogram frames. The reconstructed DICOM slices were converted to the NIFTI format with size 344x344x127 (voxel size: 2.09 × 2.09 × 2.03 mm^3^) for each volume. A 5-mm FWHM Gaussian post-filter was applied to each 3D volume. All 3D volumes were temporally concatenated to form a 4D (344 × 344 x 127 × 356) NIFTI volume. A guided motion correction method using simultaneously acquired MRI images was applied to correct motion during the PET scan. We retained the 225 16-sec volumes commencing from the 30-minute time point, which matched the start of the BOLD-fMRI EPI acquisition, for further analyses. A single static PET image was derived from the sum of the 16-sec volumes. The 225 PET volumes were motion-corrected (FSL MCFLIRT); the mean PET image was brain extracted and used to mask the 4D data. The fPET data was further processed using a spatio-temporal gradient filter to estimate the short-term change in glucose uptake from the cumulative glucose uptake that was measured.

### Motion parameters

**Fig. 2:**
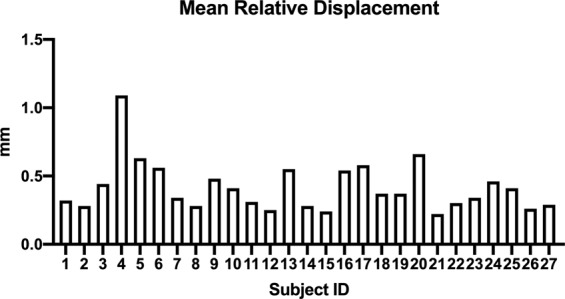
Mean relative displacement (mm) for translational motion parameters for each subject.

For the PET data, translational motion parameters for each subject were summarised by the mean distance for each direction (x, y, z) along timepoints (225 frames) for each subject, and are shown in Fig. 2. Across subjects, average mean framewise translational motion was 0.41 mm; maximum was 1.09 mm.

### Plasma radioactivity curves

**Fig. 3:**
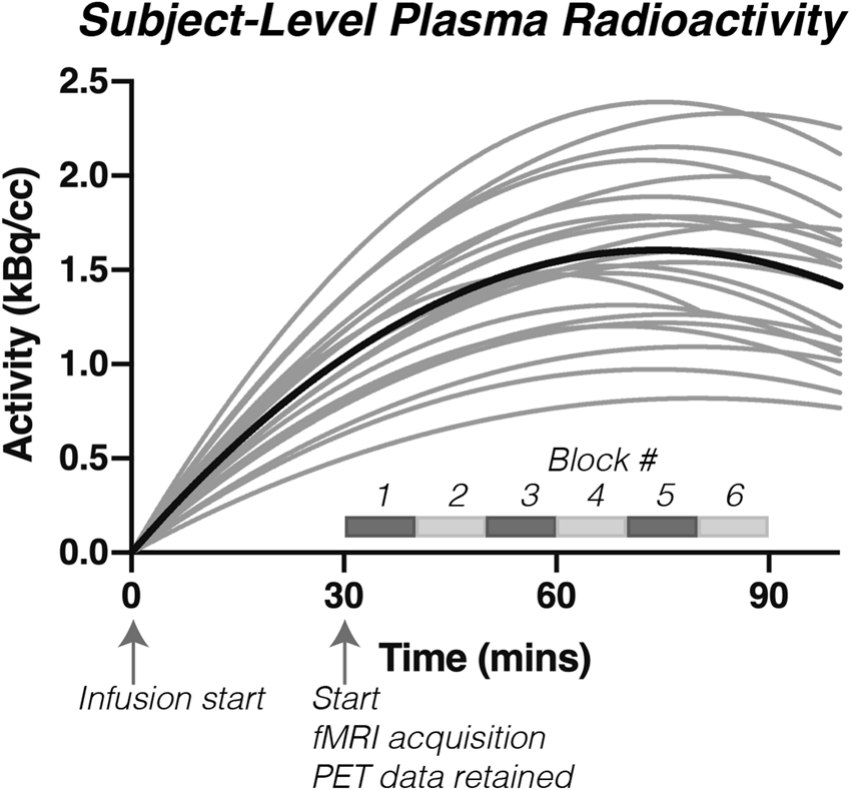
Plasma radioactivity curves for each individual subject. A 2^nd^ order polynomial was fit to the blood samples for each subject (shown in grey). The group average is shown plotted in black. Samples were not obtainable for 5 individuals.

Interpolated plasma radioactivity curves are shown in Fig. 3. Consistent with the known dynamics of the constant infusion approach^14^, plasma radioactivity increased throughout the scan, reaching a peak just prior to the end of the scan (90 mins post-infusion). After the cessation of the infusion (i.e., the last measurement point in Fig. 3), the available radioactivity declines.

### Stability of the fPET connectome over the scan period

**Fig. 4:**
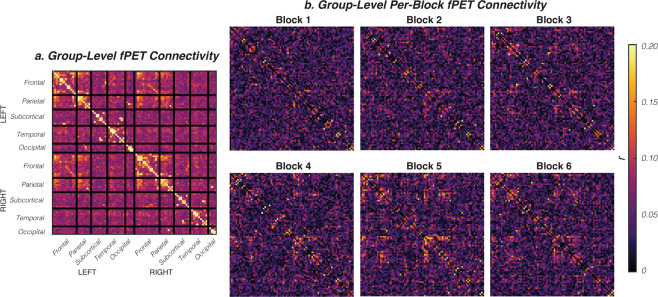
(**a**) Group-average fPET connectivity matrix, as reported in Jamadar *et al*., 2020. (**b**) Stability of fPET connectivity matrix over the six experimental blocks. Refer to Fig. 1 for the experimental design.

The fPET technique relies upon constant infusion radiotracer administration. The influence the administration procedure has on the obtained images is a matter of current investigation e.g.,^13,19^. Consequently, here we report the stability of the fPET connectome over six consecutive 10-min periods (Fig. 4), corresponding with the 6 BOLD EPI blocks (Fig. 1). As can be seen from Fig. 4, the fPET connectivity structure, characterised by primarily fronto-parietal connectivity, becomes evident around the time of the peak of plasma radioactivity (i.e., block 5). This suggests that the earlier time points contribute less to the final obtained fPET connectome structure.

### Effects of spatio-temporal filtering on fPET connectivity

**Fig. 5:**
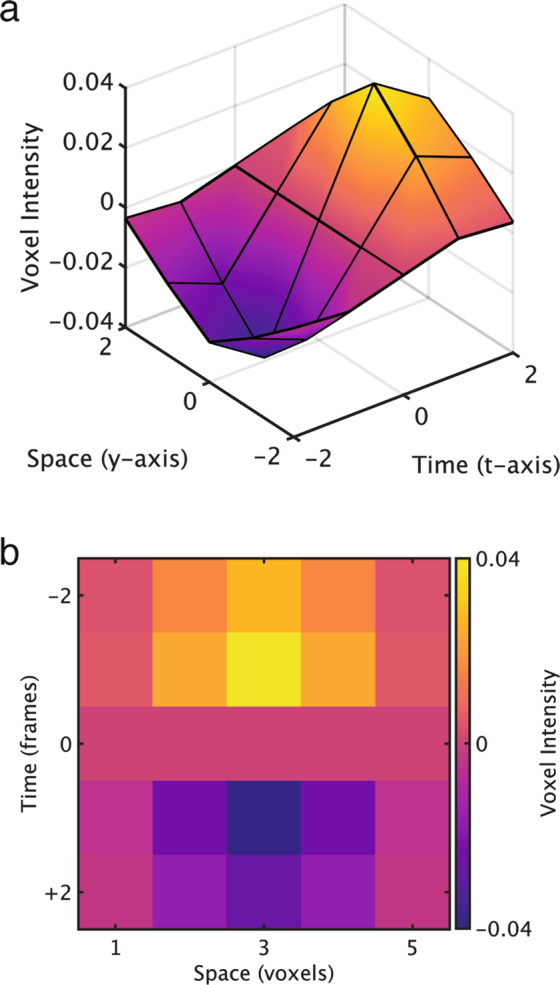
Voxel weights for the spatiotemporal filter, depicted along the y-axis and the time axis. The filter is symmetrical in the x and z axis directions.

The spatiotemporal filter was defined as the convolution of a 3-dimensional Gaussian filter in the spatial domain and a 1-dimensional Gaussian filter in the time domain, with the standard deviation being one voxel for the spatial Gaussian voxel and two frames for the temporal Gaussian (Fig. 5). The spatiotemporal convolution was further modified to give negative weights on prior frames (negative time) and zero weights for the current frame.

**Fig. 6:**
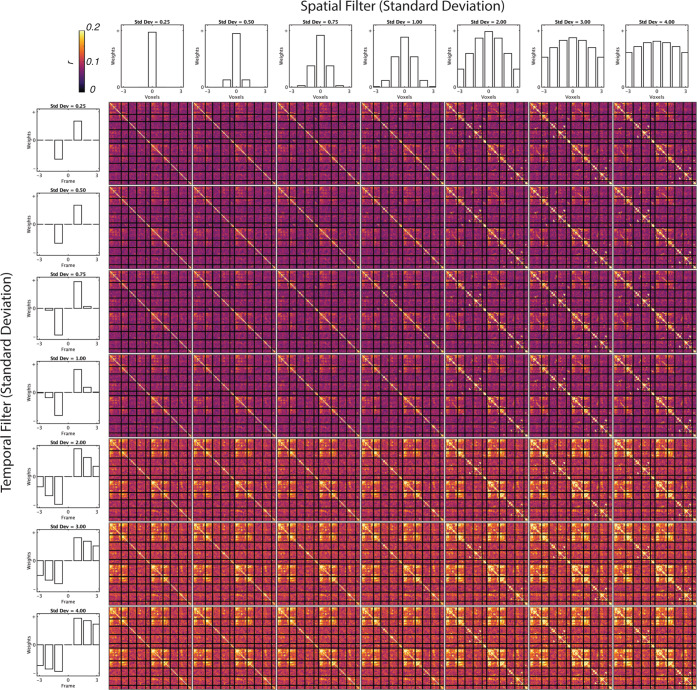
Variability of fPET connectivity matrix over a range of temporal (t) and spatial (s) filter widths.

We determined the consistency of metabolic connectivity across a range of both temporal and spatial standard deviations, up to three frames and three voxels, respectively. Figure 6 shows the variability of fPET connectivity over a range of spatial and temporal widths. We found highly consistent metabolic connectivity across a range of standard deviations. We have previously reported resting-state fPET connectivity with one voxel spatial gaussian and two frames temporal gaussian^20^. This set of parameters provided the least amount of smoothing whilst preserving the fidelity of the observed connectivity. The main trends we observed as we explored parameter values were an increase in correlation strength with higher spatial standard deviation and an increase in mean connectivity with higher temporal standard deviation.

The spatial filter is intended to de-noise the volumes by reducing non-spatially correlated noise whilst averaging spatially correlated signals. At higher standard deviation values, the filter may begin averaging signals across ROIs. We truncated the spatio-temporal filter to a 7 × 7 × 7 × 7 voxel spatio-temporal window to avoid an artificial correlation between spatially adjacent ROIs. In the temporal domain, high values correspond to estimates of the gradient at a longer timescale. In the extreme, this approximates static connectivity as the timescale approaches that of the entire session, i.e., change in activity between the start and end of the experiment. It is possible that the global increase in connectivity, shown as a yellow-hue in the lower frames of Fig. 6, are beginning to be contaminated by these longer time-period effects. Further explorations of parameter values should be performed to assess the dependence of network correlations as a function of the spatial filter kernel, and mean connectivity as a function of temporal filtering.

### Spatial variability of fMRI and fPET images

**Fig. 7:**
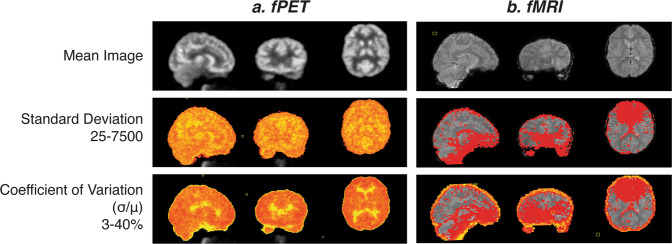
Raw images for one individual subject showing signal intensity variation across the brain. Top panel shows mean image across a 10-minute run; middle panel shows standard deviation across the same run; and lower panel shows the coefficient of variation (standard deviation divided by the mean) across the run.

fMRI and fPET connectomes showed a low region degree (i.e., little correlation with other brain regions) in subcortical regions^20^. These observations contrast with the known high degree of inter-connectedness of the subcortical areas with the rest of the brain: the cortico-basal ganglia-cerebellar (Middleton and Strick^40^; Bostan and Strick)^41^ and cortico-thalamic circuits^42^. These results are likely attributable to the reduced sensitivity of signal detection in midbrain areas for both PET and fMRI. BOLD-fMRI echo-planar images show systematic image artefacts in midbrain areas^43^ and susceptibility artefacts at air-tissue interfaces^44^. These effects are evident in the variability maps of raw echo-planar images acquired in this study (Fig. 7). The PET images have lower signal detection in the midbrain, due to the higher attenuation of gamma rays emitted from deep brain regions. The attenuation correction corrects the signal loss but cannot fix the lower signal to noise ratio in the midbrain region of the images. We observed systematically lower signal to noise ratio and high signal variability in brain regions near the ventricles (Fig. 7). The low inter-connectedness of the FDG-fPET and BOLD-fMRI measures in the subcortical areas may be attributable to the lower neuronal signals detected in these areas.

## Usage Notes

Effective sharing of research products means that data, processing methods, workflows, and tools are made available, so that they can be reused and that published findings can be reproduced. Equally important is that data should be published with integration and reuse in mind, especially when using artificial intelligence and machine learning approaches. The data can be interpreted in new ways and new knowledge attained^45^. The FAIR principles^46^ have been developed to ensure that the scientific research results are **f**indable, **a**ccessible, **i**nteroperable and **r**eusable, for both human analysis and machine analysis. Brain imaging research is in the vanguard of neuroscience research disciplines advocating open science and compliance with the FAIR principles. Neuroimaging data can make an important contribution to building a multiscale, comprehensive, and dynamic understanding of the structure and function of the nervous system.

The primary motivation behind making the Monash rsPET-MR data publicly available is to facilitate research aiming to understand the relationship between glucose metabolism and haemodynamic signals arising from neural activity in the human brain. In particular, the data provides a multitude of opportunities to investigate the dynamic nature of metabolic and haemodynamic measures of resting-state brain activity. To our knowledge, the availability of simultaneously acquired fMR and fPET data is rare. There are only a small number of biomedical imaging research facilities that have the requisite imaging technology and expertise to obtain comparable data.

The Monash rsPET-MR dataset has significant re-use value. One example includes quantitative exploration of network parameters and their influence on the obtained metabolic connectome. Our initial analysis of stationary fPET metabolic connectivity^20^ used an established method for the estimation of functional connectivity in the BOLD-fMRI literature^47,48^. However, dynamic estimates of brain connectivity take into account the temporal fluctuations of functional connectivity, since the temporal ordering of a time series of functional brain images is important^49^. Further analyses could investigate dynamic connectivity approaches, including sliding-time window approaches and models of switching between microstates^48^. The fPET images were reconstructed with 16-sec acquisition duration. While other groups have reported results using an acquisition duration of 12-sec^19^, solutions are needed for the methodological and analytical challenges in order to use FDG-fPET as a robust index of dynamic metabolic connectivity with the reduced signal-to-noise associated with shorter frames.

The Monash rsPET-MR dataset could also be re-used to develop new image processing and analysis strategies for simultaneous fMRI/fPET data. As a relatively new imaging method, tools for validating fPET data, including pre-processing and signal detection optimisation, are unavailable publicly. Several research groups have developed bespoke data analysis environments and customised analytical tools which provide a starting point for further tools development. An important technical limitation is that processing pipelines for FDG-fPET data are immature compared to the pre-processing procedures for BOLD-fMRI data. The dataset provides opportunities for further work on validating acquisition parameters, data preparation, and signal detection optimisation, in addition to the recent techniques associated with radiotracer administration e.g.,^13,19^, attenuation correction^50^, motion correction^51^ and data analysis^18,29^.

## Data Availability

Scripts used to insert required metadata into the published BIDS dataset are freely available at https://github.com/MonashBI/Monash_rsPET-MR_prep.
